# Psychometric evaluation of the simplified Japanese version of the Athens Insomnia Scale: The Fukushima Health Management Survey

**DOI:** 10.1111/jsr.12771

**Published:** 2018-10-12

**Authors:** Hajime Iwasa, Yoshitake Takebayashi, Yuriko Suzuki, Akiko Yagi, Wen Zhang, Mayumi Harigane, Masaharu Maeda, Tetsuya Ohira, Hirooki Yabe, Seiji Yasumura, the Mental Health Group of the Fukushima Health Management Survey

**Affiliations:** ^1^ Fukushima Medical University Fukushima Japan; ^2^ Japan Agency for Medical Research and Development Chiyoda‐ku Japan

**Keywords:** community dwellers, experiencing disaster, insomnia, scale development

## Abstract

We investigated the psychometric properties of the simplified Japanese version of the Athens Insomnia Scale (AIS‐SJ) using baseline data from the Fukushima Health Management Survey. Data from 22 878 men and 27 669 women aged 16 years and older were analysed (*M*_age_ = 52.9 ± 18.6). Participants lived in the Fukushima evacuation zone and experienced the Great East Japan Earthquake. The AIS‐SJ was used to assess participants’ insomnia symptoms, and its validity was examined by administering the Kessler 6‐item Psychological Distress Scale (K6) and assessing education, self‐rated health and disaster‐related experiences. A confirmatory factor analysis revealed that the two‐factor model was a better fit than the one‐factor model. The AIS‐SJ and its subscales had acceptable reliability (Cronbach's alpha, 0.81). Test of measurement invariance confirmed strict invariance across groups for the participants’ characteristics of gender and mental illness history, but not for participants’ age. AIS‐SJ scores exhibited a near‐normal distribution (skewness, 0.45; kurtosis, −0.89). There were significant age differences only among women, and gender differences in AIS‐SJ scores with small effect sizes. The AIS‐SJ scores had weak‐to‐moderate correlations with mental illness history, bereavement, experiencing the tsunami, experiencing the nuclear power plant incident, housing damage and losing one's job (polyserial correlations, 0.36, 0.17, 0.13, 0.18, 0.13, and 0.15, respectively), and strong correlations with self‐rated health (polyserial correlation, 0.51), psychological distress (*r*_s_, 0.60) and post‐traumatic stress disorder (*r*_s_, 0.60). The AIS‐SJ is a useful instrument for assessing community dwellers’ insomnia symptoms.

## 1 INTRODUCTION

On 11 March 2011, a massive earthquake and tsunami struck Japan (the Great East Japan Earthquake), leading to the Fukushima Daiichi nuclear power plant (NPP) incident (Yabe et al., 2014; Yasumura et al., 2012). Consequently, there has been an urgent need to assess mental health problems, including post‐traumatic stress disorder (PTSD), depression, anxiety and insomnia, among evacuees both within and outside of Fukushima prefecture to provide them with the relevant support and to accumulate scientific evidence for assisting future disaster survivors in Japan.

Those who experience disasters are likely to have insomnia symptoms (Kawano et al., 2016). The symptoms can result from (a) psychological problems that are caused by disasters (e.g., depression, anxiety and PTSD) and (b) mental and physical exhaustion as a result of long‐term evacuation. Because of the specific reasons related to this disaster (e.g., fear of radioactivity from the NPP incident, exhaustion from long‐term evacuation, and anxiety about the future), most residents in this region reported disturbed sleep (Iwasa et al., 2016).

There are urgent and long‐term needs to assess insomnia states among those who experienced disasters, including detecting and responding to early mental health problems, because insomnia frequently occurs in the primary stages of mental illness (Taylor, Lichstein, Durrence, Reidel, & Bush, 2005) and there can be subsequent effects of insomnia on health (Grandner, Hale, Moore, & Patel, 2010; Javaheri & Redline, 2017; Yaffe et al., 2011). Thus, we need a validated and simplified instrument for assessing insomnia.

Several measures exist to assess sleep problems in epidemiological samples. To date, the Pittsburgh Sleep Quality Index (PSQI, Doi et al., 2000) assesses sleep quality, the Athens Insomnia Scale (AIS; Okajima, Nakajima, Kobayashi, & Inoue, 2013) assesses insomnia, the Ford Insomnia Response to Stress Test (Nakajima et al., 2014) assesses vulnerability to insomnia, and the Epworth Sleepiness Scale (Takegami et al., 2009) assesses daytime sleepiness. All have been used in previous studies among Japanese community dwellers.

A previous study (Okajima et al., 2013) evaluated the psychometric properties of the AIS with 640 adults (including 477 outpatients with insomnia and 163 healthy individuals). The results revealed a two‐factor structure: nocturnal sleep problem (“Nocturnal”), items 1–5; and daytime dysfunction (“Daytime”), items 6–8. Acceptable internal consistency for the total score and both subscales, concurrent validity with external criteria using other insomnia scales (PSQI, Doi et al., 2000; and Insomnia Severity Scale, Manber et al., 2008), and known‐group validity for insomnia patients were confirmed.

We conducted a longitudinal survey to examine the mental health of community dwellers each year after the Fukushima NPP incident. To lessen their burden, we developed and implemented a simplified scale based on the AIS (the Simplified Japanese version of AIS: AIS‐SJ). However, as the psychometric properties of this scale have not been fully reported yet (e.g. reliability, validity, score distribution and age and gender differences), evaluating those properties is needed for proper use.

We sought to identify gender and age differences in AIS‐SJ scores. There is robust evidence that women are more likely to develop sleep problems (Kessler et al., 2012; Matsumoto, Yamaoka, Inoue, & Muto, 2014; Yoshioka et al., 2012) than men. Therefore, we expected that women would tend to score higher on the AIS‐SJ than men. Meanwhile, there have been no consistent findings regarding age differences in sleep problems (Kessler et al., 2012; Matsumoto et al., 2014); therefore, we sought to explore any age‐related differences in the AIS‐SJ among community dwellers in Japan.

To examine the scale's validity, we performed correlation analyses between the AIS‐SJ scores and various external criterion variables. First, because insomnia symptoms show high lifetime comorbidity with psychiatric disorders (Sbarra & Allen, 2009; Taylor et al., 2005), we assumed that individuals with a history of mental illness would have higher AIS‐SJ scores. Similarly, AIS‐SJ scores would likely be related to psychological distress; therefore, we administered the Kessler 6‐item Screening Scale for Psychological Distress (K6), which has been widely used in community surveys to assess psychological distress (Furukawa, Kessler, Slade, & Andrews, 2003). Additionally, AIS‐SJ scores would likely be related to PTSD. Sleep problems are well known to correlate with PTSD symptoms (Germain, Hall, Krakow, Katherine Shear, & Buysse, 2005); in fact, the DSM‐IV criteria for PTSD include symptoms of insomnia (American Psychiatric Association, 1994) and the scale for assessing PTSD itself contains items on disturbed sleep (Posttraumatic Stress Disorder Checklist–Stressor Specific Version [PCL‐S]; Iwasa et al., 2016; Weathers, Litz, Huska, & Keane, 1994). As such, we expected that insomnia would be related to PTSD.

Second, we expected insomnia symptoms to influence physical health status; indeed, sleep problems are associated with an increased risk of physical illness. Previous studies have reported that sleep problems are associated with risks of chronic disease (Javaheri & Redline, 2017; Kessler et al., 2012), as well as future risk of cognitive disorders (Yaffe et al., 2011) and mortality (Grandner et al., 2010). Therefore, we investigated the relationship between the AIS‐SJ and self‐rated health, which we used as an indicator of physical health status.

Finally, there is little doubt that the events caused by the Great East Japan Earthquake (the tsunami, the NPP incident and bereavement because of these disasters) were extreme stressors. Given that previous studies have reported significant relationships between PTSD symptoms and tsunamis (Heir et al., 2011), NPP incidents (Bromet & Havenaar, 2007) and bereavement (Zhang, Shi, Wang, & Liu, 2011), and that there is a close relationship between PTSD symptoms and insomnia, we expected that individuals who had experienced these events would have more sleep problems than those who had not. Additionally, individuals who experienced acute life changes such as housing damage and losing their jobs because of the disaster would have experienced levels of stress and anxiety that would also spark insomnia symptoms. As such, significant correlations were expected between AIS‐SJ scores and each event.

In sum, we examined the psychometric properties of the AIS‐SJ using baseline data from the Fukushima Health Management Survey conducted in 2012. Specifically, we determined the (a) frequencies of responses to the AIS‐SJ items, (b) factor structure, (c) reliability, (d) measurement invariance, (e) score distribution, (f) age and gender differences in AIS‐SJ scores, and (g) validity of the AIS‐SJ.

## 2 METHODS

The data for this study were drawn from a longitudinal study monitoring the mental health status of evacuees of the Fukushima Daiichi NPP incident (i.e. the Fukushima Health Management Survey). A detailed description of this survey is provided elsewhere (Yabe et al., 2014; Yasumura et al., 2012). In this study, we analysed cross‐sectional data from the first wave of data in 2012.

### 2.1 Participants

This study was approved by the Ethics Committee of Fukushima Medical University (No. 1,316). Participants included the entire population of community dwellers who lived in the evacuation zone designated by the government (*n* = 210 189) and were aged 16 years or older as of 11 March 2011. The participants’ residential information was obtained from the municipal resident registration files. These 180 604 residents were invited to participate via a mail survey, and 73 569 residents responded (response rate, 40.7%). Among them, 23 776 were excluded from the analyses for the following reasons: 138 questionnaires were incomplete and 22 884 had missing values for an insomnia scale item. The present analyses included data from 50 547 individuals (22 878 men and 27 669 women). Table 1 shows the participants’ characteristics.

**Table 1:** Participants’ characteristics (*n* = 50 547)

Participants’ characteristics	Mean (*SD*) or *n* (%)
Age, mean (*SD*)	52.9 (18.6)
Gender (women), *n* (%)	27,669 (54.7)
Education (compulsory), *n* (%)	10,993 (21.7)
Self‐rated health (poor), *n* (%)	8,880 (17.6)
Mental illness (yes), *n* (%)	2,676 (5.3)
Psychological distress, mean (*SD*)	6.1 (5.7)
PTSD, mean (*SD*)	32.2 (14.6)
Experiencing the earthquake (yes), *n* (%)	48,856 (96.7)
Experiencing the tsunami (yes), *n* (%)	9,949 (19.7)
Experiencing the nuclear power plant incident (yes), *n* (%)	26,037 (51.5)
Bereavement (yes), *n* (%)	9,390 (18.6)
Housing damage (yes), *n* (%)	7,308 (14.5)
Losing a job (yes), *n* (%)	16,867 (33.4)

Psychological distress was assessed using the Japanese version of the K6 scale. PTSD, post‐traumatic stress disorder, which was assessed using the Japanese version of the Post‐traumatic Stress Disorder Checklist–Stressor Specific Version (PCL‐S).

### 2.2 Measurements

The AIS‐SJ comprised one item measured with a 4‐point Likert‐type scale (i.e. #5 sleep quality: 1 = satisfied, 2 = slightly dissatisfied, 3 = quite dissatisfied, 4 = very dissatisfied or have not slept at all) and seven yes/no type response items (i.e. #1 sleep induction, #2 awakening during the night, #3 awakening in the early morning, #4 total sleep duration insufficiency, #6 well‐being during the day, #7 functioning capacity during the day, and #8 sleepiness during the day). The seven dichotomized items were modified from the original 4‐point Likert‐type items in the AIS. The item “sleep quality” was dichotomized into two categories: “1 = satisfied and 2 = slightly dissatisfied”, and “3 = quite dissatisfied and 4 = very dissatisfied or have not slept at all.” Item scores were summed to provide a total score (range, 0–8), with higher scores reflecting a higher level of insomnia.

Additionally, self‐rated health (1 = very good to 5 = very poor) was used to assess participants’ physical health. Psychological distress was measured using the K6 (Furukawa et al., 2003), with higher scores reflecting greater distress. Using the PCL‐S (Iwasa et al., 2016; Weathers et al., 1994), with higher scores reflecting a greater number of PTSD symptoms, we asked participants about their experiences with the Great East Japan Earthquake, including the earthquake, the tsunami and the NPP incident. We also assessed educational attainment, history of mental illness (yes/no), experiencing the earthquake (yes/no), experiencing the tsunami (yes/no), experiencing the NPP incident (yes/no), bereavement (yes/no), having housing damage (yes/no) and losing a job (yes/no) because of the Great East Japan Earthquake. Participants who had experienced the NPP incident included those who had heard the explosions from the Fukushima Daiichi NPP that occurred between 12 and 15 March 2011.

### 2.3 Data analyses

A confirmatory factor analysis (CFA) was conducted to examine the factor structure of the AIS‐SJ. A polychoric correlation matrix was applied for the CFA, using robust weighted least squares (WLSMV) as the estimator because we analysed the ordinal scale of the items. We assessed two models: the one‐factor model and the two‐factor model. The two‐factor model included the two subscales: Nocturnal (items 1–5) and Daytime (items 6–8). Indexes of model fit including the root mean square error of approximation (RMSEA) and the comparative fit index (CFI) were used. According to previous studies, model fit was deemed “adequate” or “excellent” when RMSEA ≤0.08 or ≤0.06 and CFI ≥0.90 or ≥0.95, respectively (Hu & Bentler, 1999).

Multi‐group analyses of CFA were performed to evaluate measurement invariance across groups in three participants’ characteristics (i.e. age [younger (16–64 years) vs. older (≥65 years)], gender [men versus women] and history of mental illness [experienced versus none]). The index of model fit of RMSEA and CFI was used to evaluate invariance (ΔRMSEA ≤0.015; ΔCFI ≥−0.01; Chen, 2007). To show that the AIS‐SJ scores have the same meaning across groups, the measurement invariance tests were performed in a hierarchical manner: invariance constraint of factor structure was executed among the groups (e.g. men and women; Model 1); then, the additional invariance constraint of equivalent factor loadings and residuals were added for strict invariance (Model 2). We referred to a statistical package manual regarding measurement invariance for categorical variables and used an option command for measurement invariance in Mplus (Analysis option: MODEL = CONFIGURAL/SCALAR; Muthén & Muthén–, 1998).

Furthermore, the Cronbach's alpha coefficients for the eight‐item total score and each subscale of the AIS‐SJ were calculated as indices of scale reliability. Descriptive statistics were calculated, and distributions were plotted. We also performed a two‐way analysis of variance (ANOVA) to examine gender‐ and age‐based differences in the AIS‐SJ. We demonstrated statistical values for ANOVA, including *F* values, degrees of freedom, *p*‐values and effect sizes (partial η^2^). Interpretations regarding effect size of the main effect and interaction term in ANOVA correspond to previously stated criterion; coefficients of approximately 0.0099 are weak, 0.0588 are moderate and 0.1379 are strong (Cohen, 1988).

For validity testing, nine external criteria were used: psychological distress (K6), PTSD (PCL‐S), history of mental illness, self‐rated health, experiencing the tsunami, experiencing the NPP incident, bereavement, housing damage and losing one's job. Because of non‐normal distributions in psychological distress and PTSD, Spearman's rank correlation coefficients were calculated. Additionally, polyserial correlation coefficients were calculated for history of mental illness, self‐rated health, experiencing the tsunami, experiencing the NPP incident, bereavement, housing damage and losing one's job because these external variables were all categorical (ordinal) variables, and polyserial correlation coefficients are more desirable for correlation analyses. Interpretations regarding effect size of correlation coefficients corresponded to previously stated criterion (Cohen, 1988; i.e. correlations of approximately 0.1 are weak, 0.3 are moderate and 0.5 are strong).

All probability values were two‐tailed. We used IBM SPSS Statistics version 22 (IBM Corp., Armonk, NY, USA) and Mplus version 8 (Muthén & Muthén–, 1998) for the analyses.

## 3 RESULTS

The response frequencies, as well as the mean scores and standard deviations for each item on the AIS‐SJ, are shown in the Appendix 1.

The CFA showed the following results: the one‐factor model had few acceptable model fit values (χ^2^ = 10,348.2, *df* = 20, *p* < 0.01, RMSEA = 0.10, CFI = 0.95). In contrast, the two‐factor model had adequate fit values (χ^2^ = 3,609.3, *df* = 19, *p* < 0.01, RMSEA = 0.06, CFI = 0.98). Another analysis using the full information maximum likelihood method (*n* = 73 431) induced a similar result to the above analysis using the complete data (*n* = 50 547); therefore, we retained the two‐factor model using the complete dataset (*n* = 50 547) (Table 2). Table 2 shows the standardized factor loadings and inter‐factor correlations in the CFA using the two‐factor model. The Cronbach's alpha coefficients for all eight items and for the AIS‐SJ subscales were 0.81 (all eight items), 0.80 (Factor 1) and 0.76 (Factor 2).

**Table 2:** Factor structure and loadings on the simplified Japanese version of the Athens Insomnia Scale (AIS‐SJ) (confirmatory factor analysis, *n* = 50 547)

Item	F1: Nocturnal	F2: Daytime
1. Sleep induction	0.74	
2. Awakening during the night	0.73	
3. Awakening in the early morning	0.71	
4. Sleep duration	0.80	
5. Sleep quality	0.87	
6. Well‐being during the day		0.91
7. Functioning during the day		0.91
8. Sleepiness during the day		0.62

Inter‐factor correlation was 0.77. Model fit: root mean square error of approximation = 0.06; comparative fit index = 0.98.

Table 3 shows the results of examining measurement invariance. Regarding configural invariance, adequate fit indices in Model 1 were displayed (RMSEA: 0.061 for gender, 0.061 for age, 0.063 for history of mental illness; CFI: 0.984 for gender, 0.985 for age, 0.981 for history of mental illness), which supports the configural invariance across groups in the three characteristics of participants. In the next step, the results supported strict invariance for only two characteristics of participants (ΔRMSEA between Model 2 and Model 1: 0.005 for gender, 0.018 for age, −0.002 for history of mental illness; ΔCFI between Model 2 and Model 1: −0.005 for gender, −0.013 for age, −0.001 for history of mental illness). The strict invariance for age was not confirmed (ΔRMSEA between Model 2 and Model 1: 0.018; ΔCFI between Model 2 and Model 1: −0.013).

**Table 3:** Measurement invariance for the simplified Japanese version of the Athens Insomnia Scale (AIS‐SJ) across participants’ characteristics (*n* = 50 547)

	Chi‐squared	*df*	RMSEA	ΔRMSEA	CFI	ΔCFI
Gender: men = 22,878 versus women = 27,669						
Model 1: configural invariance	3,616	38	0.061	–	0.984	–
Model 2: strict invariance		42	0.066	0.005	0.979	−0.005
Age: younger = 35,960 versus older = 14,587						
Model 1: configural invariance	3536	38	0.061	–	0.985	–
Model 2: strict invariance	6619	42	0.079	0.018	0.972	−0.013
History of mental illness: experience with = 2,676 versus none = 46,513						
Model 1: configural invariance	3720	38	0.063	–	0.981	–
Model 2: strict invariance	3893	42	0.061	−0.002	0.980	−0.001

RMSEA, root mean square error of approximation; CFI, comparative fit index.

Table 4 shows the descriptive statistics of the AIS‐SJ and its subscale scores. Note that the distribution of scores was near normal (skewness, 0.45; kurtosis, −0.89).

**Table 4:** Descriptive statistics of scores on the simplified Japanese version of the Athens Insomnia Scale (AIS‐SJ) and its subscales (*n* = 50 547)

Scales	Skewness	Kurtosis	Mean	95% confidence interval	Standard deviation	Median
Total (0–8)	0.45	−0.89	3.06	3.04–3.08	2.44	3
Nocturnal (0–5)	0.45	−0.99	1.93	1.92–1.94	1.64	2
Daytime (0–3)	0.54	−0.11	1.12	1.11–1.13	1.12	1

The mean (standard deviation) of the AIS‐SJ total scores was 3.06 (2.44) among all participants, 3.01 (2.43) in younger adults, 3.18 (2.47) in older adults, 2.80 (2.41) in men and 3.27 (2.44) in women. Two‐way ANOVAs (with age group and gender as factors) were performed. Means (standard deviations) are shown in each cell of Table 5. As a result of the two‐way ANOVAs (with age group and gender as factors), because the interaction term for AIS‐SJ score was significant, *F*(1, 50,543) = 32.06, *p* < 0.01, partial η^2^ = 0.00063, we analysed the simple main effects in the AIS‐SJ score. The simple main effect of age was not significant for men, indicating that younger men had similar scores to older men, *F*(1, 50,543) = 0.99, *p* = 0.319, partial η^2^ = 0.00002; however, the simple main effect of age was significant for women, indicating that older women had higher scores than younger women, *F*(1, 50,543) = 88.70, *p* < 0.01, partial η^2^ = 0.00175. The simple main effect of gender was significant for younger and older adults, indicating that women had higher scores than men: *F*(1, 50,543) = 242.41, *p* < 0.01, partial η^2^ = 0.00477; *F*(1, 50,543) = 277.89, *p* < 0.01, partial η^2^ = 0.00547.

**Table 5:** Age and gender differences in scores on the simplified Japanese version of the Athens Insomnia Scale (AIS‐SJ) and its subscales (*n* = 50,547)

Scales	Younger (aged 16–64 years)		Older (aged 65 years or older)	
	Men	Women	Men	Women
Total (0–8)	2.79 (2.42)	3.19 (2.41)	2.82 (3.39)	3.49 (2.51)
Nocturnal (0–5)	1.77 (1.61)	1.96 (1.62)	1.84 (1.61)	2.29 (1.71)
Daytime (0–3)	1.02 (1.11)	1.23 (1.12)	0.98 (1.08)	1.20 (1.15)

Means (standard deviations) were shown in each cell. As a result of two‐way analyses of variance (with age group and gender as factors), interaction terms for AIS‐SJ and Nocturnal scores were significant (*F*(1, 50,543) = 32.06, *p* < 0.01, partial η^2^ = 0.00063; *F*(1, 50,543) = 64.28, *p* < 0.01, partial η^2^ = 0.00127). Main effects of age and gender for Daytime score were significant (*F*(1, 50,543) = 7.86, *p* < 0.01, partial η^2^ = 0.00016; *F*(1, 50,543) = 371.94, *p* < 0.01, partial η^2^ = 0.00731).

As the interaction term for the Nocturnal subscale score was significant, *F*(1, 50,543) = 64.28, *p* < 0.01, partial η^2^ = 0.00127, we analysed the simple main effects for the Nocturnal subscale. The simple main effect of age was significant for both men and women, indicating that older adults had significantly higher scores than younger adults: *F*(1, 50,543) = 9.42, *p* < 0.01, partial η^2^ = 0.00019; *F*(1, 50,543) = 228.84, *p* < 0.01, partial η^2^ = 0.00451. The simple main effect of gender was significant for both younger and older adults, indicating that women had significantly higher scores than men: *F*(1, 50,543) = 127.45, *p* < 0.01, partial η^2^ = 0.00252; *F*(1, 50,543) = 279.89, *p* < 0.01, partial η^2^ = 0.00551.

The main effects of age and gender were significant for the Daytime subscale score, indicating that younger adults had higher scores than older adults, *F*(1, 50,543) = 7.86, *p* < 0.01, partial η^2^ = 0.00016, and that women had higher scores than men, *F*(1, 50,543) = 371.94, *p* < 0.01, partial η^2^ = 0.00731.

The validity analyses revealed that there were significant relationships between the AIS‐SJ and all criterion variables. Specifically, AIS‐SJ scores were relatively weakly associated with experiencing the tsunami, experiencing the NPP incident, bereavement, housing damage and losing one's job. Moderate correlations were found between AIS‐SJ scores and history of mental illness. Strong correlations were found between AIS‐SJ scores and self‐rated health, psychological distress and PTSD. The results across subscales were similar (see Table 6).

**Table 6:** Correlation of the simplified Japanese version of the Athens Insomnia Scale (AIS‐SJ) with external criterion variables (*n* = 50 547)

	Psychological distress^a^^.^ c	PTSD^a^^,^ d	Mental illness^b^	Self‐rated health^b^	Experiencing tsunami^b^	Experiencing NPP incident^b^	Bereavement^b^	Housing damage^b^	Losing job^b^
Total (0–8)	0.60	0.60	0.36	0.51	0.10	0.18	0.17	0.13	0.15
Nocturnal (0–5)	0.50	0.54	0.28	0.46	0.10	0.18	0.16	0.12	0.14
Daytime (0–3)	0.56	0.52	0.37	0.44	0.08	0.14	0.13	0.10	0.13

a Spearman's correlation coefficients were shown in each cell of psychological distress and PTSD.

b Polyserial correlation coefficients were shown in each cell of mental illness, self‐rated health, experiencing the tsunami, experiencing the NPP incident, bereavement, housing damage, and losing job. All correlation coefficients were significant (*p*_s_ < 0.001). Higher scores for insomnia scales, psychological distress, PTSD and self‐rated health indicate a poorer state in each index.

c Psychological distress was assessed using the Japanese version of the K6 scale.

d PTSD was assessed using the Japanese version of the Post‐traumatic Stress Disorder Checklist–Stressor Specific Version (PCL‐S). NPP, nuclear power plant; PTSD, post‐traumatic stress disorder.

## 4 DISCUSSION

According to the results of the CFA, the one‐factor model had a relatively poor model fit. In contrast, the two‐factor model had an adequate model fit. We decided to retain the two‐factor model because its fit indices had “excellent fit” cut‐offs described previously (Hu & Bentler, 1999). Our findings on the factor structure of the AIS‐SJ are consistent with those of a previous study that used the original Japanese version of the AIS (Okajima et al., 2013). In addition, the correlations between the two factors were strong (0.77), which also coincides with the results of a previous study (0.62) (Okajima et al., 2013).

The Cronbach's alpha coefficients for the AIS‐SJ and its subscales were high, suggesting acceptable reliability. This is consistent with a previous study, which also reported high reliability for the AIS (Okajima et al., 2013). Moreover, because of the multigroup analyses, measurement invariance on the AIS‐SJ was confirmed across groups for the participants’ characteristics of gender and history of mental illness. These results suggest that the meaning of an AIS‐SJ score was identical across groups on these variables and that the combined group analysis and comparing the scores across groups was adequate.

On the other hand, measurement invariance in the AIS‐SJ was not confirmed for the participants’ characteristics of age because the values of ΔRMSEA and ΔCFI for age (0.018 and −0.013) were larger than the criterion of the measurement invariance (ΔRMSEA <0.015 and ΔCFI <−0.01). Thus, the meaning of AIS‐SJ scores appeared not to be identical between the younger and older age groups, suggesting that the combined group analysis and comparing the scores across groups should be performed with caution. These findings may reflect a physiological change in sleep with ageing. Older people frequently realize their sleep states at night become shallower and shorter (Yaremchuk, 2018). Subsequently, older individuals may experience nocturnal awakening and early morning awakening. Namely, sleep problems may be recognized differently based on age: younger people may be more likely to recognize those sleep problems as serious when influenced by mental and physical illness. Meanwhile, older adults may be more likely to recognize those sleep problems as a phenomenon related to normal ageing processes.

There were complex patterns in the AIS‐SJ scores based on age, with no age difference in men; however, older women had higher scores than younger women. This suggests that insomnia symptoms in older individuals are more likely to be severe than those in younger individuals when assessed using the AIS‐SJ, but only among women. Regarding subscales, older adults had higher scores than younger adults on the Nocturnal subscale; however, the opposite pattern existed for the Daytime subscale. Therefore, age differences on the AIS‐SJ total score for men, which consists of these two subscales, might be counterbalanced and diminished by two such opposite age effects. Previous findings regarding age differences are discordant. A study conducted among insured Americans reported that older adults had fewer sleep problems than did younger individuals (Kessler et al., 2012). Among those who experienced a recent disaster, older age was not associated with sleep problems (Matsumoto et al., 2014). Combining our results and those of previous findings, the age‐related difference in sleep problems among community dwellers may not be significant. However, as noted above, the combined analysis of the age groups (younger versus older) and the comparison of scores across age groups should be performed with caution because strict measurement invariance on the AIS‐SJ was not confirmed for age groups.

We also found a significant gender difference in which the mean for women was higher than that for men across age groups. This accords with previous findings among community dwellers (Kessler et al., 2012; Matsumoto et al., 2014; Yoshioka et al., 2012) and suggests that women are more vulnerable to developing insomnia than men are. However, as shown above in the results section, small effect sizes were found for age and gender differences in AIS‐SJ scores. Thus, we concluded that although age and gender differences were statistically significant they were relatively weak.

The validity testing revealed that AIS‐SJ scores were significantly, but relatively weakly, correlated with experiencing the tsunami, experiencing the NPP incident, bereavement, housing damage and losing one's job. Additionally, there were moderate correlations with history of mental illness and self‐rated health, and strong correlations with psychological distress and PTSD. These results indicate that the AIS‐SJ has validity in relation to (a) psychological problems (Sbarra & Allen, 2009), (b) self‐rated health as a physical health indicator (Grandner et al., 2010; Javaheri & Redline, 2017; Kessler et al., 2012; Yaffe et al., 2011) and (c) experiencing disaster‐related events (Bromet & Havenaar, 2007; Heir et al., 2011; Zhang et al., 2011).

This study has some limitations. First, as this study was conducted in one region of Japan, the external validity of these findings may be limited. Nevertheless, this study examined the psychometric properties of the AIS‐SJ using a large sample of community‐dwelling adults (over 50 000 persons) with a broad age range (16–100 years). Second, we did not examine test–retest reliability of the AIS‐SJ; we examined only the internal consistency reliability using Cronbach's alpha coefficients. Future research should evaluate the stability of the scale by examining test–retest reliability. Finally, we could not evaluate concurrent validity through examining the association between the AIS‐SJ and the original version of the AIS‐J (Okajima et al., 2013) as a reference standard measure because we did not have data for the original version of the AIS‐J.

In conclusion, we determined the psychometric properties of the AIS‐SJ using baseline data from the Fukushima Health Management Survey. Standard psychometric methods showed a near‐normal score distribution. The AIS‐SJ had a two‐factor structure, involving measurement invariance across groups for the participants’ characteristics of gender and mental illness history, excellent reliability and good validity. We also noted gender and age differences only among women in AIS‐SJ scores. In summary, the results indicate that the AIS‐SJ is useful for assessing insomnia in community dwellers.

## CONFLICT OF INTEREST

The authors declare no conflict of interest.

## AUTHOR CONTRIBUTIONS

SY and HY initiated the study and collected the data. HI and YT analysed and interpreted the data, and HI wrote the first draft of the manuscript. All authors contributed to the manuscript and critical discussion. All of the authors read and approved the final manuscript.

## APPENDIX 1

Response frequencies of the Simplified Japanese version of the Athens Insomnia Scale (AIS‐SJ) items

**Table:**

	Satisfied	Slightly dissatisfied	Quite dissatisfied	Very dissatisfied
Sleep quality (original four choices), *n* (%)	17,994 (35.6)	22,787 (45.1)	7,563 (15.0)	2,203 (4.4)

Item	Yes (problem)	No (no problem)
1. Sleep induction, *n* (%)	20,645 (40.8)	29,902 (59.2)
2. Awakening during the night, *n* (%)	30,234 (59.8)	20,313 (40.2)
3. Awakening in the early morning, *n* (%)	17,538 (34.7)	33,009 (65.3)
4. Sleep duration, *n* (%)	19,601 (38.8)	30,946 (61.2)
5. Sleep quality (dichotomized), *n* (%)	9,766 (19.3)	40,781 (80.7)
6. Well‐being during the day, *n* (%)	15,415 (30.5)	35,132 (69.5)
7. Functioning during the day, *n* (%)	16,209 (32.1)	34,338 (67.9)
8. Sleepiness during the day, *n* (%)	25,152 (49.8)	25,395 (50.2)

“Sleep quality” was dichotomized into two categories: “1, satisfied and 2, slightly dissatisfied”; and “3, quite dissatisfied and 4, very dissatisfied or have not slept at all.”
